# A 9-year-old boy with a nonmalignant forehead tumor – a rare case of pediatric Pott’s puffy tumor

**DOI:** 10.1186/s12887-023-04117-8

**Published:** 2023-06-17

**Authors:** Jesper Amstrup, Lasse Wedege Penning, Jonas Kjeldbjerg Hansen, Ninna Brix, Graziella Andersen

**Affiliations:** 1 Department of Pediatric and Adolescent Medicine, Aalborg University Hospital, Aalborg, Denmark; 2 Department of Radiology, Aalborg University Hospital, Aalborg, Denmark

**Keywords:** Case report, Pott’s Puffy Tumor, Pediatric, Radiology, Nonmalignant tumor

## Abstract

**Background:**

Pott’s puffy tumor (PPT) is a rare and potentially deadly complication of frontal sinusitis consisting of subperiosteal abscess and osteomyelitis of the frontal bone.

**Case presentation:**

We report the case of a 9-year-old boy who presented with fever and soft tissue swelling of the forehead. Magnetic resonance imaging (MRI) depicted an abscess in the subcutaneous tissue frontally and an epidural empyema, while a cranial computed tomography (CT) scan revealed bone erosion as a sign of osteomyelitis. The patient was treated accordingly.

**Conclusions:**

This rare condition is essential to keep in mind as it needs a multidisciplinary approach and relevant imaging to start proper treatment and thus decrease the risk of intracranial complications.

## Background

Pott’s puffy tumor (PPT) is a rare diagnosis characterized by localized swelling of the forehead due to a subperiosteal abscess of the frontal sinus with underlying osteomyelitis of the frontal bone. It was first described by surgeon Sir Percival Pott as a consequence of head trauma, though the currently prominent cause is inadequately treated frontal sinusitis [1–3]. The most common clinical manifestations include headache, fever, rhinorrhea, and forehead swelling. Early diagnosis is essential as treatment should be initiated as early as possible in order to avoid intracranial complications, including epidural and subdural empyema, cerebral abscesses, meningitis, and cerebral venous thrombosis. It is an important condition to be aware of when evaluating children with frontal sinusitis not responding adequately to antibiotics [4, 5]. We report a rare case of PPT in a pediatric patient, who was diagnosed by magnetic resonance imaging (MRI).

## Case presentation

**Fig. 1:**
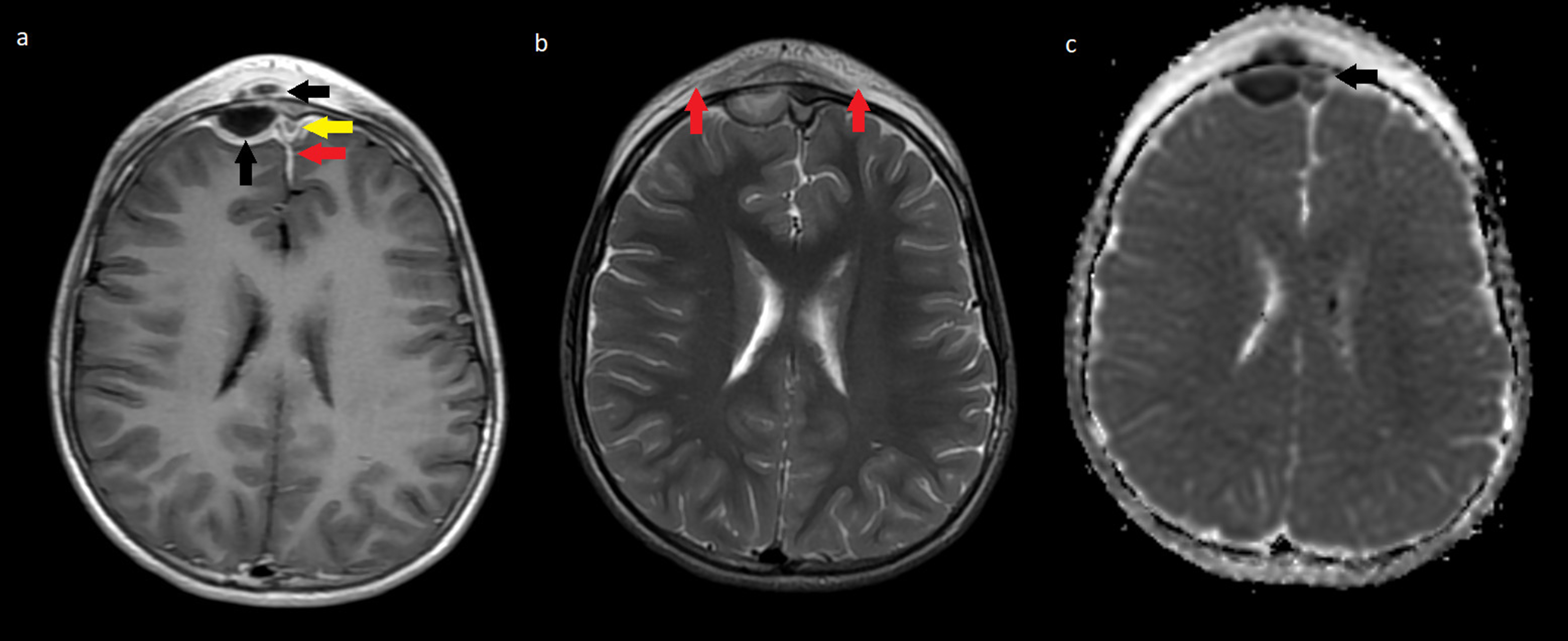
Gadolinium-enhanced magnetic resonance of the head. **a**: T1 weighted sequence with gadolinium showing a 20 × 9 mm frontal epidural collection with 2 mm wide capsular contrast-enhancement, minor local cerebral mass effect and an additional external periosteal collection measuring 8 mm with capsular contrast-enhancement suggestive of a pathological soft tissue reaction compatible with cellulitis (black arrows). There is dural enhancement frontally bilaterally and along falx cerebri frontally (red arrow). The anterior third part of the superior sagittal sinus shows wall thickening with concomitant flow obstruction as a result of reactive phlebitis due to the nearby infection (yellow arrow). A contrast-enhanced MR venogram showed no sinus thrombosis (image not included). **b**: T2 weighted sequence shows soft tissue edema frontally and no pathological signals in the brain parenchyma (red arrows). **c**: Apparent diffusion coefficient sequence showing restricted diffusion in the mentioned encapsulated collection further confirming the tentative diagnosis of abscesses and an additional similar collection positioned in the midline frontally (black arrow). No pathological signals in the brain parenchyma

**Fig. 2:**
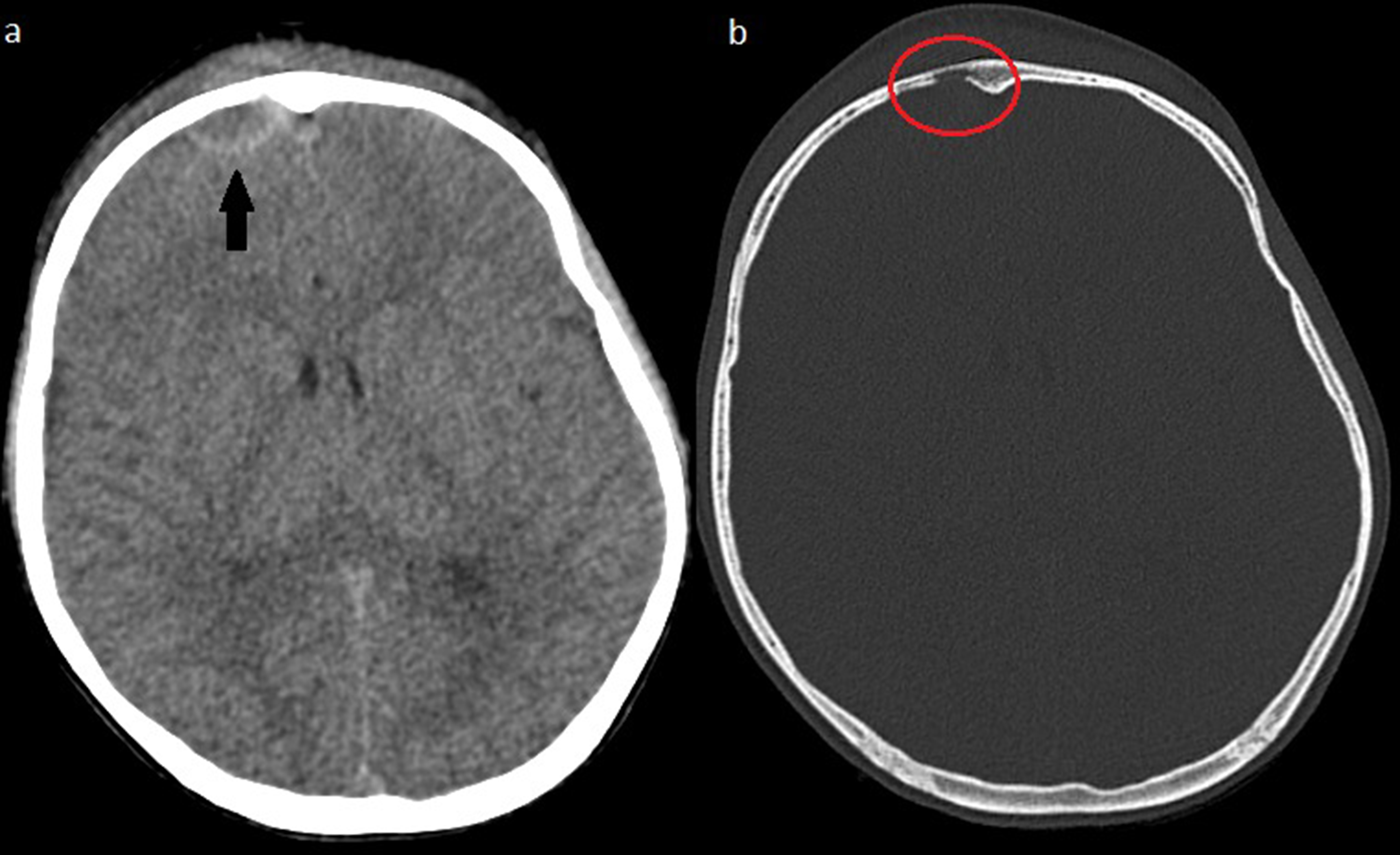
Spiral computed tomography of the head (performed after the MRI scan resulting in remainders of gadolinium contrast). **a**: An epidural 20 × 9 mm collection with capsular contrast-enhancement suggestive of an abscess and soft tissue swelling frontally (black arrow). **b**: Bone erosion internally of the frontal bone suggestive of osteomyelitis (red circle)

A previously healthy 9-year-old boy presented with a headache and incipient frontal swelling. After approximately ten days, the headache worsened, and he got a fever. He was then treated with penicillin for seven days, but four days after completion, the symptoms relapsed with increased swelling, pain behind the right eye, and a temperature of up to 40 degrees Celsius. The patient was admitted to an otorhinolaryngologist 24 days after the first onset of symptoms. On physical examination, there was swelling and pain at pressure on the right side of the forehead delimited to both eyebrows down to os nasale. There was no fluctuation, redness, or softness of the skin corresponding to the bone. Neurological examination was normal. The flexible nasoendoscopy was unremarkable, but an ultrasound depicted tissue edema without signs of abscess formation. Due to the uncharacteristic symptoms and clinical findings, a computed tomography (CT) scan of the sinuses (CTDI 1.44 mGy) was performed showing signs of bone erosions in relation to the frontal sinuses, although not visualized in its entirety. The patient was sent for evaluation at the pediatric ward. Gadolinium-enhanced MRI scan (Fig. 1) was performed, showing slight maxillary, ethmoidal and frontal sinusitis on the left side, osteomyelitis of the frontal bone, and in communication herewith an epidural and periosteal mass with capsular contrast-enhancement compatible with an abscess. Additionally, the contrast-enhanced MR venogram revealed partial obstruction in the anterior third part of the superior sagittal sinus in close relation to the abscesses due to reactive phlebitis without thrombosis. A subsequent CT scan of the cerebrum (Fig. 2, CTDI 24.30, mGy) gave a full visualization of the frontal bone erosion internally on the right side.

Blood analyses showed an increased C-reactive protein level of 29 mg/L (< 8 mg/L) and white blood cell count of 24.2 × 10^9^/L (4.5–12.5 × 10^9^/L) with a neutrophil count of 19.8 × 10^9^/L (1.8–8.9 × 10^9^/L). The patient was diagnosed with PPT and followed a prolonged course of intravenous antibiotic therapy with ceftriaxone and metronidazole. Cultures of blood yielded *Streptococcus anginosus*. The patient was consulted with the neurosurgery department finding no indication for surgical intervention due to no neurological deficits and the patient’s well-being. After one week of hospitalization the patient went home to continue the intravenous antibiotic treatment for six weeks in total with frequent consultations. On follow-up for two months, the patient was in complete remission, both clinical and radiological.

## Discussion and conclusions

We highlight a very rare case of pediatric PPT. Once the patient was admitted due to an inadequate antibiotic response, a multidisciplinary approach and collaboration between medical specialties rapidly established the diagnosis, leading to quick and efficient treatment. MRI verified the diagnosis, and the patient did not need neurosurgical intervention as he soon became fever and pain-free and had no sequelae. PPT predominantly affects adolescents due to a development peak in the vascularity of the diploic circulation and growth of the frontal sinus. Failure to recognize the diagnosis early can lead to critical progression and intracranial complications, including meningitis and intraparenchymal abscess [2, 3, 6].

The majority of cases are associated with an upper respiratory tract infection, thus different types of streptococci, staphylococci, and anaerobes are the most common organisms causing PPT [2]. In this case, blood culture yielded *Streptococcus anginosus*, which has only been described once before [3, 5, 7]. Due to the risk of polymicrobial infection, broad-spectrum antibiotics are recommended to cover a variety of aerobic and anaerobic microorganisms [2, 3].

CT and MRI scans are crucial in confirming the diagnosis of PPT when there is a clinical history of sinusitis and swelling of the forehead. On a CT scan, an easily accessible imaging modality in most cases, sinusitis, and signs of osteomyelitis in the form of bone opacification and destruction, along with potential involvement of the frontal sinuses and air-trapping in bone or soft tissue, can be seen. In this case the initial CT scan of the sinuses arouse suspicion of the diagnosis, while the later CT scan of the cerebrum was performed in order to evaluate and fully visualize the extension of the bone erosion. Incidentally, the remaining gadolinium from the MRI scan resulted in capsular contrast-enhancement of the already established abscess. MRI with gadolinium-enhancement is superior and first choice in soft tissue presentation, and therefore the golden standard in evaluating suspected intracranial complications of PPT and should always be performed if neurological symptoms are observed [8]. The different sequences can show the extent of PPT and the involvement of or effect on dural venous sinuses, cerebral parenchyma, and other soft tissues [7]. In this case, the diffusion-weighted sequences depicted an epidural abscess in the midline that was difficult to differentiate from the dural sinuses on other sequences. Furthermore, contrast-enhanced MR venogram ruled out a proper sinus venous thrombosis, a possible complication to PTT, but did visualize obstruction of flow in the anterior part of the superior sagittal sinus due to phlebitis without the need for any additional treatment. Also, MRI avoids ionizing radiation, which is especially relevant in pediatric patients.

PPT is a rare and serious complication of inadequately treated frontal sinusitis or head trauma. A multidisciplinary approach between pediatricians and radiologists ensured effective diagnosis in hospital, as MRI confirmed the diagnosis and revealed intracranial abscesses not visible on the CT scan. This rare condition is important to keep in mind as it requires an interdepartmental approach and relevant imaging to start proper treatment and thus decrease the risk of intracranial complications.

## Data Availability

Not applicable.

## List of abbreviations

MRI: Magnetic resonance imaging
CT: Computed tomography
PPT: Pott’s puffy tumor
